# Body mass index and variability in meal duration and association with rate of eating

**DOI:** 10.3389/fnut.2022.941001

**Published:** 2022-07-26

**Authors:** Stacey L. Simon, Zhaoxing Pan, Tyson Marden, Wenru Zhou, Tonmoy Ghosh, Delwar Hossain, J. Graham Thomas, Megan A. McCrory, Edward Sazonov, Janine Higgins

**Affiliations:** ^1^Department of Pediatrics, University of Colorado Anschutz Medical Campus, Aurora, CO, United States; ^2^Colorado Clinical and Translational Institute, University of Colorado Anschutz Medical Campus, Aurora, CO, United States; ^3^Electrical and Computer Engineering (ECE), The University of Alabama, Tuscaloosa, AL, United States; ^4^Department of Psychiatry and Human Behavior, Alpert Medical School of Brown University, Providence, RI, United States; ^5^Department of Health Sciences, Boston University, Boston, MA, United States

**Keywords:** eating rate, eating speed, dietary intake, food consumption, obesity, BMI

## Abstract

**Background:**

A fast rate of eating is associated with a higher risk for obesity but existing studies are limited by reliance on self-report and the consistency of eating rate has not been examined across all meals in a day. The goal of the current analysis was to examine associations between meal duration, rate of eating, and body mass index (BMI) and to assess the variance of meal duration and eating rate across different meals during the day.

**Methods:**

Using an observational cross-sectional study design, non-smoking participants aged 18–45 years (*N* = 29) consumed all meals (breakfast, lunch, and dinner) on a single day in a pseudo free-living environment. Participants were allowed to choose any food and beverages from a University food court and consume their desired amount with no time restrictions. Weighed food records and a log of meal start and end times, to calculate duration, were obtained by a trained research assistant. Spearman's correlations and multiple linear regressions examined associations between BMI and meal duration and rate of eating.

**Results:**

Participants were 65% male and 48% white. A shorter meal duration was associated with a higher BMI at breakfast but not lunch or dinner, after adjusting for age and sex (*p* = 0.03). Faster rate of eating was associated with higher BMI across all meals (*p* = 0.04) and higher energy intake for all meals (*p* < 0.001). Intra-individual rates of eating were not significantly different across breakfast, lunch, and dinner (*p* = 0.96).

**Conclusion:**

Shorter beakfast and a faster rate of eating across all meals were associated with higher BMI in a pseudo free-living environment. An individual's rate of eating is constant over all meals in a day. These data support weight reduction interventions focusing on the rate of eating at all meals throughout the day and provide evidence for specifically directing attention to breakfast eating behaviors.

## Introduction

Approximately 42% of U.S. adults meet the criteria for obesity, with the highest prevalence in non-Hispanic black adults compared with other racial/ethnic groups, significantly increasing the risk for morbidity and mortality (1, 2). Both behavioral and pharmacological weight-loss interventions are available but not optimally effective for all individuals, so the identification of alternative strategies and techniques to facilitate weight loss and maintenance are urgently needed (3). The rate of eating may be one area for investigation. Fast eating is independently associated with overweight and obesity, weight gain, and risk for health consequences, such as cardiovascular disease and type 2 diabetes (4–7).

Observational studies have found that individuals who are overweight or obese tend to eat at a faster rate than individuals who are not (8). A meta-analysis of correlational studies found that individuals who eat quickly are two times more likely to be obese (4). In contrast, another study reported no difference in the rate of eating as measured by a chewing rate between individuals with high or normal body mass index (BMI) (9). A large study of adults from the Singapore Multi-Ethnic Cohort using food frequency questionnaires to assess dietary intake found that individuals who are obese and fast eaters consumed a larger proportion of high energy intake rate foods, defined as foods that can be consumed more quickly to facilitate higher energy intake (10, 11). Indeed, energy intake is higher when foods can be eaten at a faster rate (12). However, self-reported eating rates have been found to be inaccurate (13). Thus, additional research using a high-quality methodology is needed to clarify the relationship between eating rate and weight status.

Experimental laboratory-based studies manipulating eating rate have been conducted, changing eating rate *via* different methodology, such as verbal instructions, changing food textures, and providing automated feedback (8). Overall, in laboratory conditions, faster eating rate is associated with the higher energy intake (8). During a slower eating rate condition, individuals reported greater fullness compared with their “normal” eating pace (14, 15); notably the normal eating rate group endorsed greater enjoyment and satisfaction from the meal than the slower rate group (14). Eating rate was not associated with hunger ratings at the end of the meal or hours later and did not impact subsequent *ad libitum* snack intake (8, 15).

It has been posited that an eating rate is relatively consistent within an individual but this has only been tested by examining changes in the eating rate within a single meal (16) and assessing the eating rate for the same meal across several days (17). No research to date has examined intra-individual differences in the rate of eating by meal (e.g., breakfast, lunch, and dinner). This information is important as it could help inform future behavioral interventions to focus efforts to slow eating only during meals where it would have maximal impact.

Thus, the goal of the current analysis was to evaluate associations with meal duration, rate of eating, and BMI, and to assess the consistency of meal duration and eating rate across an entire day (breakfast, lunch, and dinner). We hypothesized that higher BMI would be associated with the shorter meal duration and a faster eating rate. Further, we hypothesized that an individual's eating rate would be consistent across all meals of the day.

## Materials and methods

### Participants

Non-smoking participants aged 18–45 years (*N* = 29) who had no self-reported medical conditions that affected their ability to eat or chew food, including food allergies, were recruited using flyers posted on the University of Alabama—Tuscaloosa campus. Recruitment inclusion/exclusion criteria were intentionally left very broad to recruit a range of individuals to facilitate our associative analyses. The University of Alabama's Institutional Review Board approved the study and all individuals gave written, informed consent prior to participation. The data used for the current analysis were part of a larger study examining a non-invasive, wearable device to detect food intake. Participants consumed meals in a pseudo-free living environment while wearing the device but only weighed food records and a manual log of meal duration completed by trained research assistants were used for the purpose of the current analyses.

### Procedure

Utilizing an observational cross-sectional study design, participants came to the laboratory three times on a single day to consume breakfast, lunch, and dinner, and were instructed not to consume any food or beverages, other than water, at any other time of the day. Participants ate together in groups of three to four to simulate social eating. Breakfast took place between 7:00 and 9:00 a.m. after an overnight fast; lunch and dinner took place at 12:00–2:00 p.m. and 5:00–7:00 p.m., respectively. Participants exited the laboratory between meals and went about their usual daily activities.

For each meal, participants were given the opportunity to choose any food and beverage items in the amounts they desired from the University of Alabama Cafeteria or restaurants in the adjacent food court. A research assistant weighed each food item to the nearest 0.1 g (Touch II Ozeri, San Diego, CA, USA). Each food item was weighed separately and discretely in a private location in the cafeteria or food court; combined foods (e.g., hamburger or sandwich) were deconstructed and each element was weighed separately. After each food item was weighed and recorded for each participant, the group proceeded to the laboratory to consume their meal.

In the laboratory, as a small group, participants were instructed to eat as much or little of their meal as they desired, with no restriction on time allotted for consuming the meal. After each participant indicated that they were done with their meal and all participants left the laboratory, a research assistant weighed any leftovers on the same scale. Research assistants recorded the start and stop time for each meal the participant consumed.

### Measures

#### Demographics and BMI

A research assistant recorded the participants' date of birth and measured height and weight prior to breakfast. BMI was then calculated as weight/height (kg/m^2^).

#### Energy intake, length of meal duration, and rate of eating

A research assistant entered data from the weighed food record into Food Processor Nutrition Analysis Software (version 7.6, ESHA Research, Salem, OR) to derive the energy intake (kcal) for each eating episode. A separate member of the study team who was a trained nutritionist and did not participate in the collection of the weighed food records validated data entry from the weighed food record vs. the Food Processor entries for each food item and made corrections if any data entry errors occurred. Meal duration was calculated as the amount of time between the start and end time for each meal. The rate of eating was calculated as the total energy consumed divided by the meal duration. Values were calculated separately for each meal, and for all meals across the whole day.

### Statistical analysis

This was a secondary data analysis and, therefore, no *a priori* power analysis was conducted. The appropriateness of normality assumption for continuous variables was examined using the Shapiro–Wilk test and visual examination of residual plots for multiple linear regression. Mean, standard deviation (SD), and median and range were used to summarize normally distributed and non-normally distributed continuous variables, respectively. Categorical variables were summarized with percentage. The Spearman correlation (r_s_) was used for bivariate correlation analysis. Multiple linear regression was used to examine the association of BMI with meal duration and the rate of eating while adjusting for participant's age and sex. These two covariates were included in the model regardless of their statistical significance. No adjustment was made for the number of people eating at the same time in a group. The rates of eating and duration of meals were compared across the three meals using a linear mixed effect model with unstructured covariance to account for the heterogeneities of variance and within-participant correlation across meals. The value of *p* < 0.05 was deemed statistically significant. All analyses were performed using SAS software, version 9.4. (SAS Institute, North Carolina, USA). All data are expressed as mean ± SEM, unless otherwise indicated.

## Results

### Participant characteristics

Twenty-nine participants completed the study. Participants were 65% male and the median age was 21 years (range = 18–32 years). The reported race/ethnicity of participants was 48% white (*n* = 14), 34% Asian or Pacific Islander (*n* = 10), 7% Hispanic (*n* = 2), 7% black (*n* = 2), and 1 individual identified as other race (3%). The mean BMI was 23.3 ± 3.0 kg/m^2^ with 17% of participants (*n* = 5) meeting criteria for overweight or obesity (BMI ≥ 25 kg/m^2^).

### Associations among BMI, meal duration, and rate of eating

The total daily energy intake was 2,176 ± 650 kcal, and the total meal duration, summed across all meals, was 40.6 ± 13.8 min (Table 1). Age was significantly positively correlated with the total meal duration and the total energy intake. Sex differences were observed, with women consuming less energy over all meals relative to men (1,707 ± 368 vs. 2,423 ± 634 kcal, respectively; *p* = 0.003) and demonstrating a slower rate of eating across all meals (45.9 ±17 vs. 64.8 ± 25.1 kcal/min, respectively; *p* = 0.04) but no difference in meal duration compared with men (38.3 ± 6.4 vs. 41.6 ±14.0 min, *p* = 0.52).

**Table 1:** Energy intake, meal duration, and the rate of eating for all meals, breakfast, lunch, and dinner.

	**All participants** ***N*** = **29**	**Comparison**
**Energy intake (kcal)**		
All meals	2,176.3 ± 649.9*	Overall F test
Breakfast	615.7 ± 243.6*	Breakfast - Dinner
Lunch	742.3 ± 316.8	Breakfast - Lunch
Dinner	818.3 ± 374.1	Dinner - Lunch
**Energy density (kcal/g)**		
All meals	1.5 ± 0.5*	Overall F test
Breakfast	1.3 ± 0.6^∧^	Breakfast - Dinner
Lunch	1.7 ± 0.8*	Breakfast - Lunch
Dinner	1.7 ± 0.7	Dinner - Lunch
**Meal duration (min)**		
All meals	40.6 ± 13.8*	Overall F test
Breakfast	10.7 ± 4.2^∧^	Breakfast - Dinner
Lunch	13.0 ± 4.2*	Breakfast - Lunch
Dinner	17.0 ± 10.6*	Dinner - Lunch
**Rate of eating (kcal/min)**		
All meals	58.3 ± 24.1	Overall F test
Breakfast	61.8 ± 28.9	Breakfast - Dinner
Lunch	60.8 ± 28.8	Breakfast - Lunch
Dinner	59.2 ± 37.8	Dinner - Lunch

All values arbher M ± SD or n (%).

* Denotes p < 0.05.

∧ Denotes p < 0.01.

**Figure 1:**
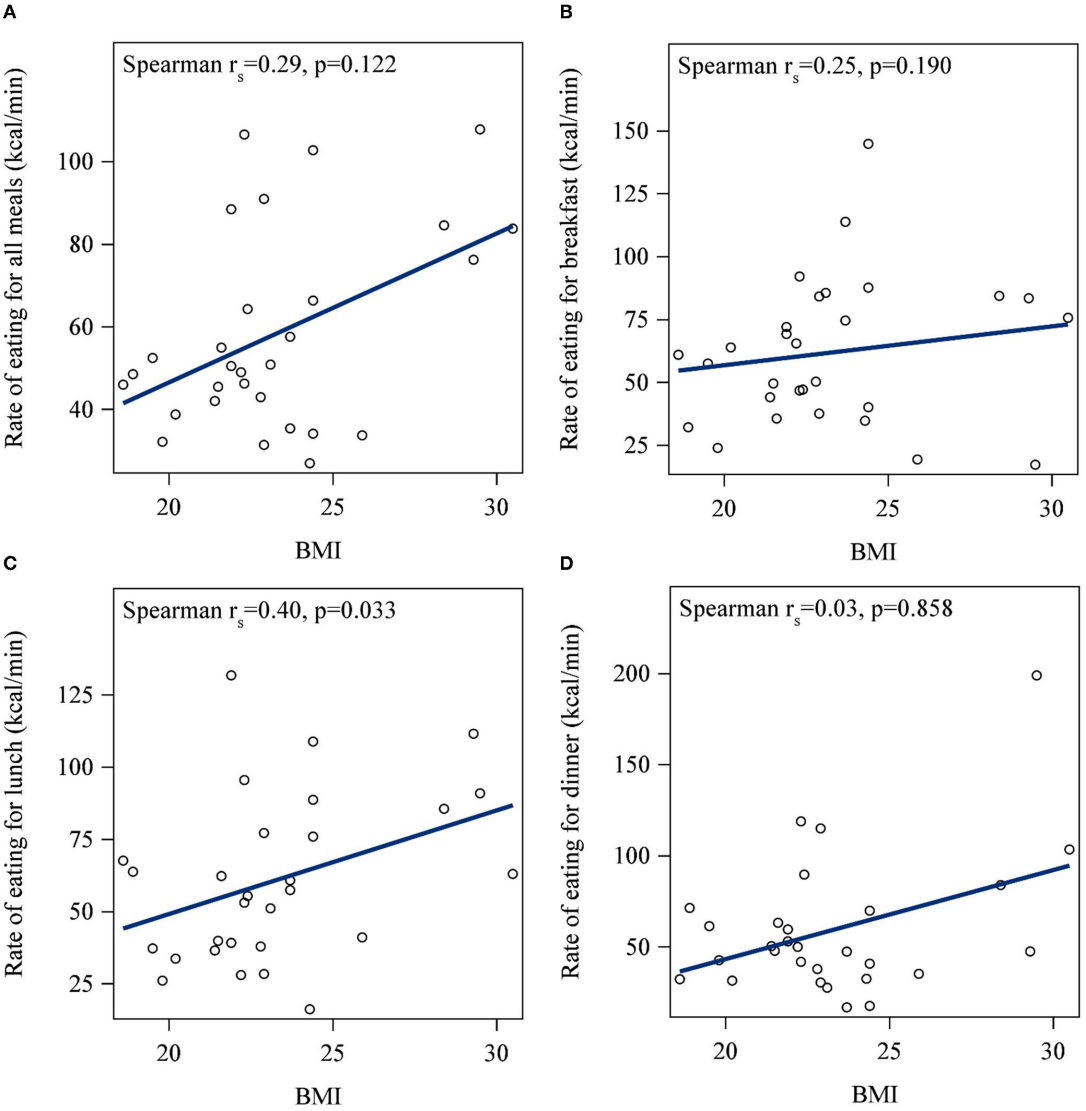
Correlations between body mass index (BMI) and meal duration for **(A)** all meals, **(B)** breakfast, **(C)**, lunch, and **(D)** dinner. No significant bivariate correlation was found between BMI and meal duration for all meals or any individual meals.

No significant bivariate correlation was found between BMI and meal duration for any individual meal (Figure 1). However, after adjusting for sex and age, shorter meal duration was associated with higher BMI at breakfast only (*p* = 0.03). Specifically, each minute less of breakfast duration was associated with a 0.32 higher BMI (Table 2). There was no association between meal duration and energy intake for any meals (*p* > 0.05).

**Table 2:** Association of BMI with meal duration and the rate of eating for breakfast, lunch, dinner, and all meals.

	**BMI and rate of eating**			
**Model**	**Independent variable**	**Coefficient (95% CI)**	**Independent variable**	**Coefficient (95% CI)**
Breakfast	Constant	23.66 (16.95, 30.38)	Constant	23.18 (15.41, 30.95)
	Male vs. Female	2.06 (−0.77, 4.88)	Male vs. Female	2.33 (−0.81, 5.46)
	Age (years)	0.07 (−0.29, 0.43)	Age (years)	−0.09 (−0.45, 0.27)
	Breakfast (min)	−0.32 (−0.6, −0.03)*	Breakfast (kcal/min)	0.01 (−0.03, 0.05)
Lunch	Constant	23.14 (15.94, 30.33)	Constant	21.44 (13.85, 29.03)
	Male vs. Female	1.6 (−1.68, 4.87)	Male vs. Female	1.76 (−1.3, 4.82)
	Age (years)	0.08 (−0.36, 0.53)	Age (years)	−0.06 (−0.4, 0.29)
	Lunch (min)	−0.22 (−0.56, 0.12)	Lunch (kcal/min)	0.03 (−0.01, 0.07)
Dinner	Constant	23.73 (16.34, 31.11)	Constant	17.36 (7.06, 27.66)
	Male vs. Female	2.46 (−0.64, 5.56)	Male vs. Female	0.16 (−3.85, 4.16)
	Age (years)	−0.09 (−0.47, 0.29)	Age (years)	0.16 (−0.29, 0.62)
	Dinner (min)	−0.01 (−0.12, 0.11)	Dinner (kcal/min)	0.04 (−0.01, 0.08)
All meals	Constant	23.43 (16.21, 30.64)	Constant	16.96 (7.43, 26.49)
	Male vs. Female	1.98 (−1.14, 5.11)	Male vs. Female	0.3 (−3.26, 3.85)
	Age (years)	0.04 (−0.38, 0.45)	Age (years)	0.12 (−0.27, 0.51)
	All Meals (min)	−0.06 (−0.16, 0.04)	All Meals (kcal/min)	0.06 (0, 0.12)*

Multiple linear regression models adjusted for participants' sex and age.

* Denotes p < 0.05.

**Figure 2:**
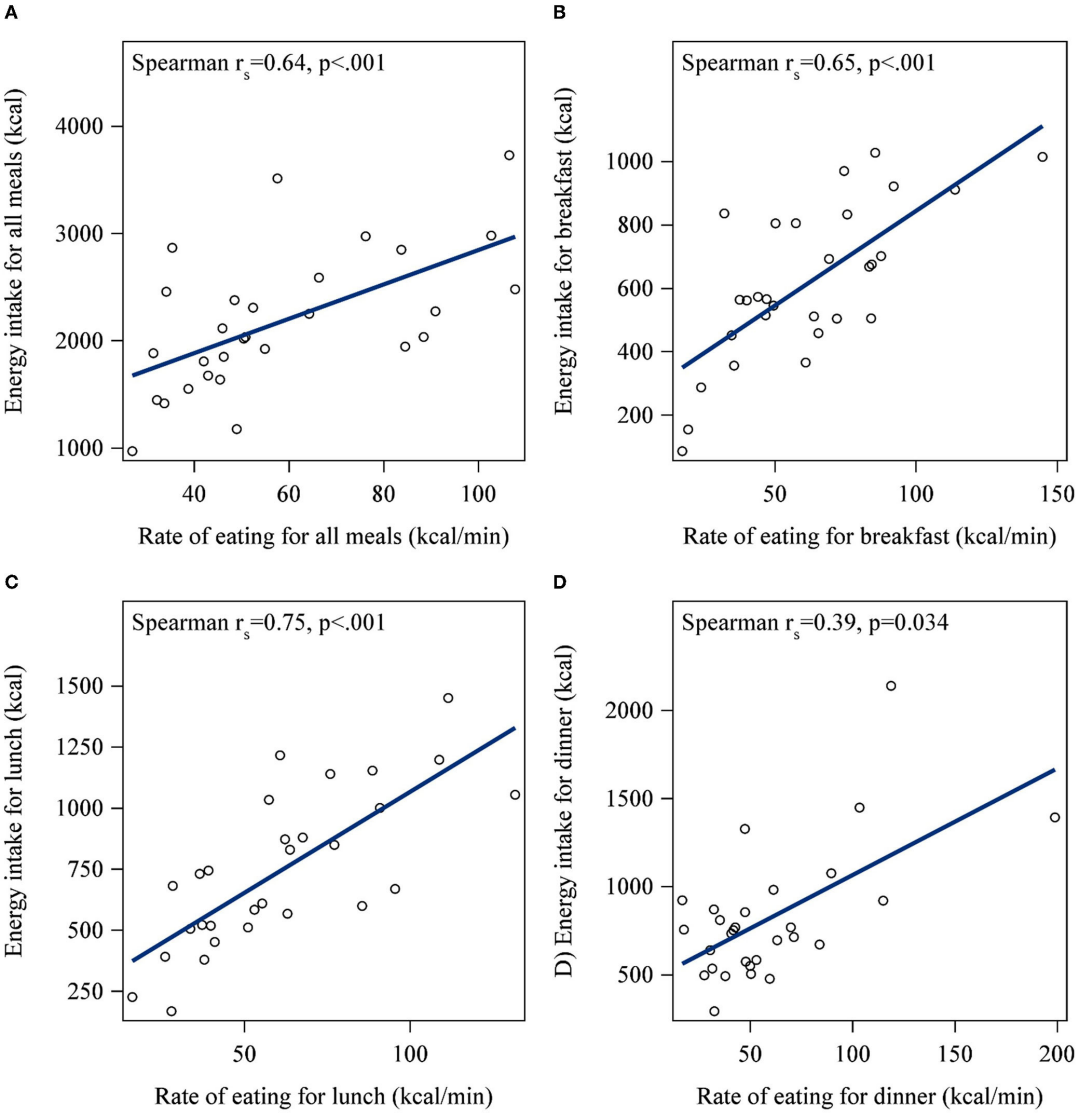
Correlations between BMI and the rate of eating for **(A)** all meals, **(B)** breakfast, **(C)** lunch, and **(D)** dinner. A higher BMI was associated with the faster rate of eating at lunch (r_s_ = 0.4; *p* = 0.03) but not breakfast, dinner, or the sum of all meals.

**Figure 3:**
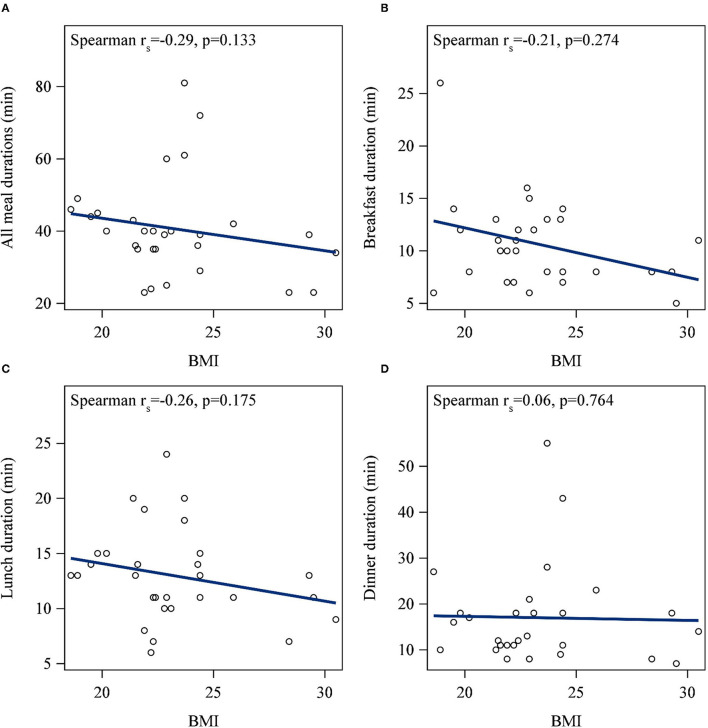
Correlations between energy intake and the rate of eating for **(A)** all meals summed, **(B)** breakfast, **(C)** lunch, and **(D)** dinner. The rate of eating was positively associated with energy intake for each meal, and the total across all meals (all *p* < 0.05).

A significant bivariate correlation was found between BMI and the rate of eating at lunch, but not breakfast or dinner (Figure 2). Specifically, higher BMI was associated with a faster rate of eating at lunch (r_s_ = 0.4; *p* = 0.03). After adjusting for sex and age, higher BMI was significantly associated with a faster rate of eating across all meals (*p* = 0.04; Table 2). The rate of eating was positively associated with energy intake for each meal, and summed across all meals (all *p* < 0.05; Figure 3).

### Intra-individual variability in energy intake, meal duration, and rate of eating across meals

**Figure 4:**
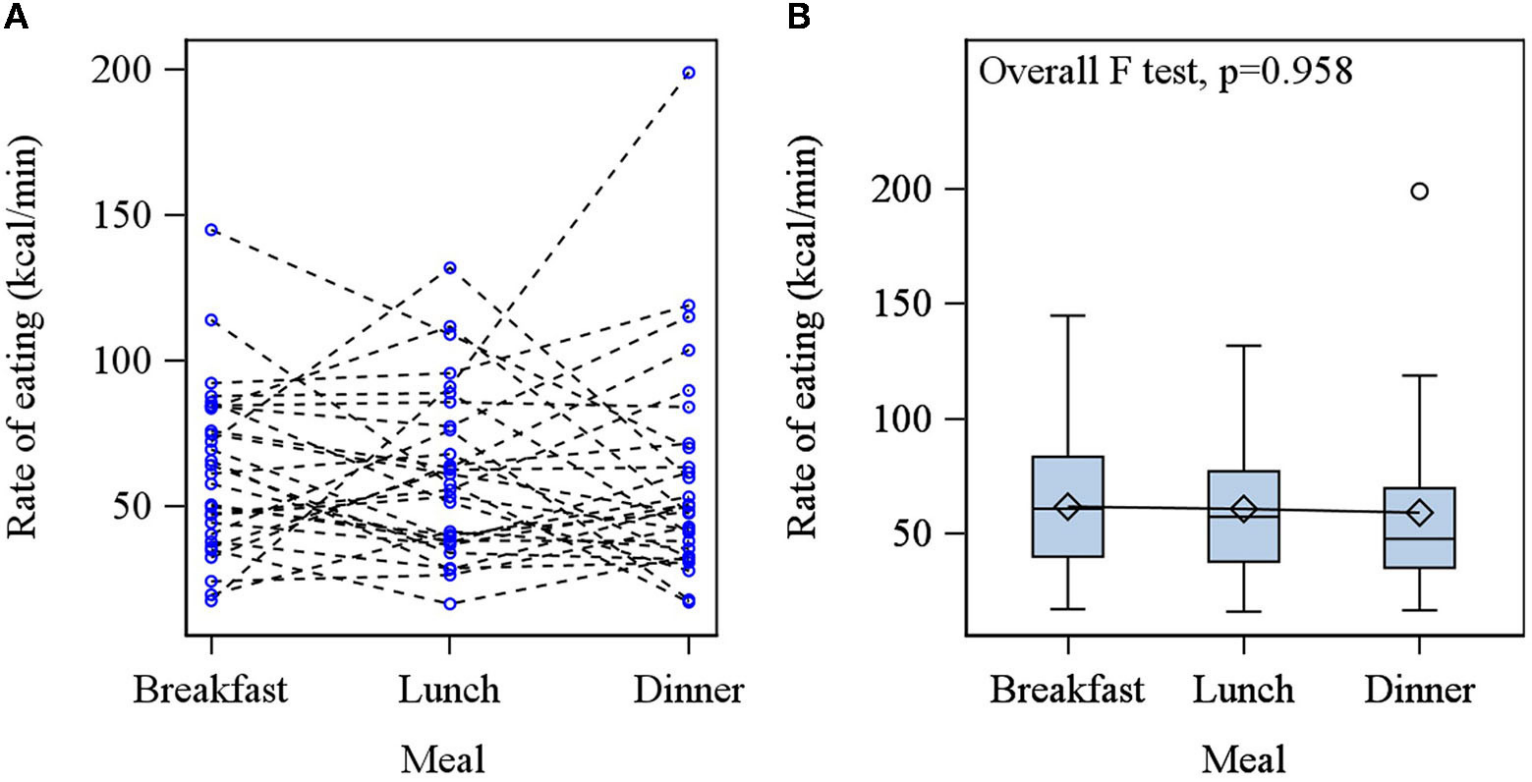
**(A)** Individual profile of the rate of eating across meals; each participant is represented by separate dotted lines. **(B)** The box and whisker plot of rate of eating at each meal. No significant difference in the rate of eating was found between breakfast, lunch, or dinner.

Individuals consumed significantly more energy at dinner compared with breakfast, with no difference in energy intake between breakfast and lunch or lunch and dinner (Table 1). The meal duration was significantly longer for dinner compared with breakfast and lunch. No significant intra-individual difference was found for the rate of eating at breakfast, lunch, and dinner (*p* > 0.05; Figures 4A,B), analyzed per individual or on a group level.

## Discussion

This study is the first to demonstrate consistency in an individual's eating rate over a full day of meals. This adds to the existing literature that examined eating rate within a single meal (16) or the same meal across several days (17). In this sample of healthy individuals in a pseudo free-living environment, a higher BMI was associated with shorter breakfast duration, and a faster objectively-measured rate of eating across all meals. Moreover, a faster rate of eating was associated with greater energy intake for all meals. These findings are broadly consistent with the extant literature showing that faster eating is associated with overweight/obesity and higher energy intake (4, 8, 11, 18–20) with two notable exceptions. The first is a prior study that found no difference in the chewing rate by BMI (9). However, this study did not measure energy intake and only examined two food types, pizza and rice, which are relatively homogeneous and may not be sufficient to observe differences. In a recent study designed similarly to ours, wearing an automated device that captures food intake in a free-living setting, the reduced eating rate was associated with higher energy intake only in normal weight, but not in obese, participants (21). The authors conclude that this finding may be related to those with obesity not wanting to record all food intake and, therefore, not wearing the camera for all meals and snacks. Additionally, this wearable device was very large and obvious during use, being strapped to the torso, which may have decreased compliance. Our study has overcome this limitation, as recommended by the authors, by using a more discrete micro-camera attached to eyeglasses (21). Finally, this study did not utilize images captured by the wearable device to estimate energy intake. Rather energy intake was measured *via* 24-h dietary recalls. This methodology caused a mismatch between device-captured and self-reported occurrence of eating episodes, only 32% of self-reported intake episodes were captured by the wearable device, and that makes intake data difficult to align and interpret.

An aggregate calculation from 23 observational studies of BMI and eating rate found that fast eaters had a 1.78 kg/m^2^ higher BMI than slow eaters (4), which may be sex dependent with fast eating women having 1.13 kg/m^2^ vs. fast eating men having 0.29 kg/m^2^ higher BMI than healthy weight controls (22). In this study, we did not have the power to assess sex differences but, unlike most previous studies that had all or predominantly female participants, our study had a greater proportion of male than female participants and also showed that higher rate of eating was associated with higher BMI. Clearly, larger prospective studies are required to assess sex-based relationships between eating rate and BMI. Overall, cross-sectional studies from Asia, Holland, and New Zealand concur in their findings that a higher self-reported eating rate is associated with higher BMI (6, 7, 22, 23). These studies all utilize self-reported eating rate which has never been validated using a broad range of food items with different textures and properties that may effect the rate of chewing and eating (24). Therefore, our study, in a pseudo free-living population consuming dozens of different choices that can affect the eating rate and our objective measurement of dietary intake over different meals, enhances the existing literature by confirming previous reports that a higher rate of eating is associated with higher BMI.

Conversely, a systematic review of 22 studies showed that experimentally slowing the eating rate lowered energy intake regardless of the type of manipulation used to alter the eating rate (8). However, in a well-controlled study feeding a mixed meal lunch, experimentally slowing the eating rate decreased total energy intake in those with normal weight but not those with overweight or obesity (25). Prior research has shown that individuals with obesity have larger bite size, less chewing per bite, and greater energy intake during eating occasions (24). Faster rate of eating has previously been found to be associated with weight gain, obesity, and related comorbidities, such as cardiovascular disease and type 2 diabetes (4–7). In a large cross-sectional study in middle-aged women, a faster rate of eating was significantly associated with higher body weight and BMI but did not predict future weight gain (23).

The rate of eating was associated with BMI across all meals. One mechanism behind the association may be *via* an impact on satiety (8). Specifically, eating rate may directly impact satiety hormones: slower eating results in greater ghrelin suppression after meals (14). The rate of eating may effect stomach distention due to the rate of gastric emptying, which may, in turn, influence feelings of satiety. Increased oral sensory exposure is associated with lower energy intake suggesting that the magnitude of exposure to taste may impact satiety (8). Finally, slower eating is associated with a greater number of sips, bites, or chews of each unit of food, and there may be a learned association between the number of sips, bites, or chews and feelings of satiety (8).

Another potential mechanism underlying these relationships may be the characteristics of food choices. Individuals with overweight/obesity that self-reported as faster eaters consumed a greater proportion of energy from higher energy-intake-rate foods (e.g., softly textured and energy dense) compared with individuals without overweight/obesity (11). Food texture influences oral processing, including bite size and the amount of chewing, which in turn impacts the rate of eating (24). A diet consisting of higher energy-intake-rate foods may be associated with the faster speed of eating as well as greater weight gain and risk for obesity.

Interventions to reduce eating rate may be beneficial for individuals with obesity but ideally need to be easily incorporated into everyday life. A mindful eating intervention had no effect on food consumption (26), suggesting that an intervention specifically targeting the duration of the meal and/or rate of eating is needed. Other strategies that have been suggested for slowing the eating rate include chewing longer, taking smaller or fewer bites, eating with a smaller utensil, and pausing between bites, but these strategies require additional examination of effectiveness and sustainability (17, 27, 28). Manipulating food texture, such as reducing the amount of caloric beverages consumed with a meal, may also have a similar effect such that thicker and chewier consistency foods require more chewing, forcing an individual to eat more slowly (29).

Several limitations of the current study should be acknowledged. While the study design tried to mimic the free-living environment, the assessment of eating did take place in a laboratory, so it may not be representative of typical behaviors. Moreover, only 1 day of eating was assessed, which may not be reflective of habitual eating habits. Finally, the sample size was relatively small and consisted of healthy individuals so generalizability may be limited. However, advantages of the current study over the existing literature include use of a gold standard weighed food record for the measurement of energy intake, assessment of all meals in a day rather than only a single meal, and direct observation in a well-controlled setting rather than relying on self-report.

## Conclusion

Shorter breakfasts and a faster rate of eating across all meals were associated with higher BMI in a sample of healthy individuals in a pseudo free-living environment. Taken together with previous intervention studies, these data support weight reduction interventions focusing on the rate of eating at all meals throughout the day and provide evidence for specifically directing attention to breakfast eating behaviors for weight loss and maintenance. Future research is needed on the impact of these eating behaviors on body composition longitudinally, and to understand other individual characteristics that may impact eating rate.

## Data availability statement

The raw data supporting the conclusions of this article will be made available by the authors, without undue reservation.

## Ethics statement

The studies involving human participants were reviewed and approved by University of Alabama Institutional Review Board. The patients/participants provided their written informed consent to participate in this study.

## Author contributions

JH, MM, and ES designed the study. TG, DH, and TM collected the data. ZP and WZ analyzed the data. JH, MM, JT, TM, and SS provided data interpretation. SS wrote the manuscript draft. All authors reviewed and edited the manuscript and approved the final manuscript.

## Funding

Research reported in this publication was supported by the National Institutes of Health through the National Institute of Diabetes and Digestive and Kidney Diseases award numbers R21DK085462 and R01DK100796 and the National Center for Advancing Translational Sciences award number U01TR002535.

## Conflict of interest

JT is a consultant and advisory board member for the Lumme Health, Inc. and the Medifast Inc. The remaining authors declare that the research was conducted in the absence of any commercial or financial relationships that could be construed as a potential conflict of interest.

## Author disclaimer

The content is solely the responsibility of the authors and does not necessarily represent the official views of the National Institutes of Health.

## Publisher's note

All claims expressed in this article are solely those of the authors and do not necessarily represent those of their affiliated organizations, or those of the publisher, the editors and the reviewers. Any product that may be evaluated in this article, or claim that may be made by its manufacturer, is not guaranteed or endorsed by the publisher.
